# Modulation of Gut Microbiota in the Management of Metabolic Disorders: The Prospects and Challenges

**DOI:** 10.3390/ijms15034158

**Published:** 2014-03-07

**Authors:** Omotayo O. Erejuwa, Siti A. Sulaiman, Mohd S. Ab Wahab

**Affiliations:** Department of Pharmacology, School of Medical Sciences, Universiti Sains Malaysia, 16150 Kubang Kerian, Kelantan, Malaysia; E-Mails: sbsamrah@kb.usm.my (S.A.S.); msuhaimi@kb.usm.my (M.S.A.W.)

**Keywords:** gut microbiota, gut microbiome, microbes, prebiotics, probiotics, fiber, antimicrobial agents, bariatric surgery, metabolic diseases, obesity, diabetes mellitus

## Abstract

The gut microbiota plays a number of important roles including digestion, metabolism, extraction of nutrients, synthesis of vitamins, prevention against pathogen colonization, and modulation of the immune system. Alterations or changes in composition and biodiversity of the gut microbiota have been associated with many gastrointestinal tract (GIT) disorders such as inflammatory bowel disease and colon cancer. Recent evidence suggests that altered composition and diversity of gut microbiota may play a role in the increased prevalence of metabolic diseases. This review article has two main objectives. First, it underscores approaches (such as probiotics, prebiotics, antimicrobial agents, bariatric surgery, and weight loss strategies) and their prospects in modulating the gut microbiota in the management of metabolic diseases. Second, it highlights some of the current challenges and discusses areas of future research as it relates to the gut microbiota and metabolic diseases. The prospect of modulating the gut microbiota seems promising. However, considering that research investigating the role of gut microbiota in metabolic diseases is still in its infancy, more rigorous and well-designed *in vitro*, animal and clinical studies are needed.

## 1. Introduction

In the last few decades, the prevalences of metabolic disorders, such as obesity, dyslipidemia, insulin resistance, and diabetes mellitus have been on the rise. These metabolic diseases, which are associated with greater risk factors for other diseases, contribute considerably to increased incidence of cardiovascular disease [1,2]. Even though several pharmacological agents are available for the management of metabolic diseases, only a fraction of treated patients respond effectively to treatment regimens [3]. Therefore, the metabolic derangements, which often characterize these diseases, predispose patients to cardiovascular disease and increased mortality. Until now, besides genetic susceptibility, environmental factors (such as physical inactivity and unhealthy dietary habits) were believed to play a predominant role in the etiology of metabolic diseases [4]. However, recent findings indicate that altered gut microbial composition and diversity may also contribute to the increased global incidence of metabolic disorders. The human gut, the largest microbial reservoir in the body, harbors about 10^13–14^ microorganisms (predominantly bacteria) [5]. These microorganisms are collectively referred to as microbiota, while their collective genomes constitute what is known as the microbiome. Most of these gut microorganisms reside in the large intestine (colon), which contains an estimated 10^11–12^ bacterial concentrations per gram of content [6]. These gut microbes play a number of physiological roles such as digestion, metabolism, extraction of nutrients, synthesis of vitamins, prevention against colonization by pathogens, and immunomodulation [7,8].

Over the past couples of years, the gut microbiota has been linked to some pathophysiological states, such as inflammatory bowel disease and colon cancer [9]. With recent research and high-throughput technologies, the roles of gut microbes have been extended to many other diseases, such as metabolic disorders [10]. The aim of this review is to underscore the prospects and challenges of modulating the gut microbiota in the management of metabolic disorders. The first part presents some of the mounting evidence implicating the role of gut microbiota in the pathophysiology of metabolic diseases. The various approaches (such as probiotics, prebiotics, antimicrobial agents, surgery, and weight loss strategies) of modulating the gut microbiota and their prospects in the management of metabolic diseases are highlighted and discussed in the second part of the review. The third part emphasizes some of the challenges confronting research into the role of gut microbiota in metabolic diseases and also posits areas of future research.

## 2. Role of Gut Microbiota in Metabolic Disorders

The role of the gut microbiota was thought to be restricted to the gut until now. However, in the past few decades, research has revealed that the gut microbiota plays a vital role in pathophysiology of metabolic disorders especially obesity and diabetes [11]. The use of germ-free mice provides early evidence in support of gut microbially derived nutrients and energy. The germ-free mice needed to consume about 30% more calories than their conventionally raised counterparts in order to sustain a similar body weight [12]. Later evidence emanates from the study of Backhed and colleagues who reported that germ-free mice had substantially less body fat even with greater food consumption than conventionally raised mice [13]. The study showed that conventionalization of germ-free mice with gut microbiota resulted in 60% increase in total body fat. The conventionalized mice also demonstrated many characteristic features of metabolic diseases, such as increased intestinal absorption of monosaccharides, insulin resistance, enhanced triglyceride synthesis, increased expression levels of lipogenetic enzymes (acetyl-CoA carboxylase and fatty acid synthase), and increased leptin level [13]. Additional evidence implicating the role of gut microbiota in metabolic diseases was later reported. For instance, unlike conventional mice, germ-free mice fed a high-fat, carbohydrate-rich diet did not develop obesity [14]. Compared to lean mice, obese mice were found to have a 50% decrease in the quantity of Bacteroidetes, an increased abundance of Firmicutes, more quantities of cecal microbial fermentation products, acetate and butyrate, and less fecal energy contents [15,16]. Unlike in the lean mice, obese gut microbiome is rich in genes involved in energy harvest and metabolism. It has also been found that obese gut microbiome phenotype is transferable to germ-free mice via transplantation of gut microbiota [17].

Similar findings have also been observed in obese humans [18]. Mice fed a high-fat diet (HFD) were recently shown to have increased levels of endotoxin, proinflammatory cytokines, macrophage infiltration, inflammation, and dysregulated gut microbiota (induced growth of Enterobecteriaceae and increased Firmicutes to Bacteriodetes ratio) [19]. Similarly, obese humans have decreased gut bacterial diversity, greater abundance of Proteobacteria (known for its pro-inflammatory role) and reduced Bacteroidetes/Firmicutes ratio [20,21]. Analysis of the phenotypes of obese and normal weight/lean microbiome has shown that obesity is associated with different bacterial genera and species. For instance, obesity is associated with increased abundance of Lactobacillus and Staphylococcus, while normal weight is associated with Bifidobacterium, Methanobrevibacter, and Bacteroidetes [22,23]. Compared to insulin-sensitive obese subjects, insulin resistance is associated with differential gut microbiota profile in insulin-resistant obese subjects [24]. The obese subjects also have high abundance of H_2_-producing microbes such as the Firmicutes and Prevotellaceae [25].

These bacteria are capable of metabolizing complex and indigestible polysaccharides to readily absorbable monosaccharides and short-chain fatty acids (SCFAs)—acetate, butyrate, and propionate [26]. The SCFAs have been shown to bind and activate two G-protein-coupled receptors, GPR41 and GPR43, of the gut epithelial cells [27]. Activation of these receptors leads to peptide YY (PYY) secretion, which suppresses gut motility and retards intestinal transit. This in turn will augment absorption of nutrients including monosaccharides derived from the indigestible carbohydrates. By this mechanism of SCFA-linked G-protein-coupled receptor activation, the gut microbiota may markedly contribute to increased nutrient uptake and deposition and, thus, contributes to metabolic disorders. The presence of gut microbiota is associated with increased exposure to gut microbially derived products such as lipopolysaccharides (LPSs). The LPSs (commonly found in Gram-negative bacteria’s outer membrane) are endotoxins that cause metabolic endotoxemia, which is characterized by the release of proinflammatory molecules [28]. The role of LPSs is also implicated in the etiology of metabolic diseases including insulin resistance and diabetes [29]. The LPS-induced signaling cascade via Toll-Like Receptor 4 (TLR4) has been shown recently to impair pancreatic β-cell function via suppressed glucose-induced insulin secretion and decreased mRNA expression of pancreas-duodenum homebox-1 (PDX-1) [30]. Compositional changes in the number of Bifidobacterium, Lactobacillus, and Clostridium as well as reduced Firmicutes to Bacteroidetes ratio in gut microbiota have also been recently reported type 1 diabetic patients [31]. The study also found that bacteria involved in the maintenance of gut integrity were significantly lower in diabetic patients than in the healthy controls [31]. Similar compositional changes in intestinal microbiota have also been documented in type 2 diabetic patients [32,33]. Several other studies linking the gut microbiota to metabolic disorders, such as insulin resistance, obesity, and diabetes mellitus, have been reviewed in more details by other authors. For more recent reviews, see the following references [34–36].

The evidence shows characteristic differences between microbial community that inhabits the gut in healthy and unhealthy states especially in metabolic disorders. Studies in both rodents and humans reveal that disorders, such as overweight, obesity, insulin resistance, and diabetes mellitus are associated with altered gut microbiota. Even though there is no consensus on which particular genera or species of microbes are always correlated with metabolic disorders, reduced quantity of Bacteroidetes; and an increased abundance of Firmicutes have been found in many of the studies. These data generally implicate the role of gut microbiota in the pathophysiology of metabolic disorders. A role mediated via increased gut nutrient extraction and absorption, increased body weight and fat, increased fat synthesis and deposition, inflammation, regulation of host pathways involved in energy homeostasis, as well as other yet to be delineated mechanisms.

## 3. Modulation of Gut Microbiota in the Management of Metabolic Disorders: The Prospects

In view of evidence that links the disruption in the composition and diversity of the gut microbiota to the development of metabolic diseases, approaches aimed at restoring the gut microbiota are emerging as potential and intriguing therapy. This section examines some of these strategies and their prospects in the management of metabolic disorders.

### 3.1. Modulation with Probiotics

One approach to modulate the gut microbiota is the ingestion or administration of probiotics. Probiotics are live microorganisms, which after entering the gut can exert beneficial health effects on the host by improving its intestinal microbial balance [37]. The most commonly used probiotics are strains of Lactobacilli and Bifidobacteria. They are usually ingested as part of dietary supplements or fermented foods including yogurt. Generally, probiotics are considered safe for human ingestion with limited or no reported cases of adverse events. Data from experimental and clinical studies suggest the modulation of gut microbiota via administration of probiotics may be an effective strategy to treat metabolic diseases. In Caco-2 cell line, genetically engineered strains of *Escherichia coli* and glucose stimulated the epithelial cells resulting in the secretion of insulin and insulinotropic proteins— glucagon-like peptide-1 (GLP-1) and PDX-1 [38]. The efficacy of probiotics in ameliorating metabolic disturbances has been demonstrated in a high-fructose-fed rat model. The study found that Lr263 (a probiotic containing *Lactobacillus reuteri*) markedly suppressed the elevated levels of serum glucose, insulin, leptin, C-peptide, glycated hemoglobin, liver injury markers, and lipid profile parameters in high fructose-fed rats [39]. These effects were associated with increased number of Bifidobacterium and Lactobacillus strains. In high-fat diet-induced obese mice and streptozotocin- or alloxan-induced diabetic rats, administration of *L. plantarum*, *L. acidophilus*, or *L. casei* resulted in reduction of body weight, hyperglycemia, epididymal fat, triglyceride, insulin and leptin, lipase activity, non-esterified fatty acids, triglyceride, low density lipoprotein (LDL)-cholesterol, LDL/HDL (high density lipoprotein) ratio, and adipocyte interleukin-1beta mRNA expression [40–43]. Supplementation with this probiotic also increased HDL-cholesterol, improved immunological parameters and exerted protective effects on the pancreas, liver and kidney. Recently, other two species of Lactobacillus, *L. rhamnosus* and *L. gasseri* were shown to exert anti-diabetic and anti-obesity effects in mice fed high-fat or high-sucrose diet. These metabolic effects include attenuated weight gain, reduced serum levels of leptin and insulin, improved insulin sensitivity, increased adiponectin production, down-regulated expression of hepatic gluconeogenic genes, up-regulated expression of hepatic fatty acid oxidative genes, and increased skeletal muscle glucose transporter (GLUT)4 mRNA expression [44,45]. Probiotic supplementation also altered gut microbiota composition, improved glucose tolerance, restored the expressions of GLUT4, PPAR-gamma and lipogenic genes as well as reduced concentrations of pro-inflammatory markers such as interleukin 6 (IL-6) and TNF-alpha in high fructose-fed rats [46]. Some of the effects of probiotics administration on the composition and diversity of gut microbiota and metabolic abnormalities in rodents are presented in Table 1.

In humans, oral supplementation with probiotics helps to maintain serum insulin concentrations in pregnant women or preserves insulin sensitivity in subjects with impaired glucose tolerance or type 2 diabetes [47,48]. A clinical study that compared the effects of conventional and probiotic (containing strains of Lactobacillus and Bifidobacterium) yogurts on lipid abnormalities found that probiotic yogurt consumption reduced the concentrations of LDL cholesterol and total cholesterol, as well as ameliorated atherogenic indices (reduced ratios of LDL:HDL and total cholesterol:HDL cholesterol) in type 2 diabetic subjects [49]. Probiotic yogurt supplementation also improved glycemic control (reduced fasting blood glucose and glycated hemoglobin) in type 2 diabetic subjects [50]. The beneficial effects of consumption of multispecies probiotic supplements on insulin resistance and metabolic profiles including high-sensitivity C-reactive protein (hs-CRP) have also been reported in diabetic patients [51]. Supplementation with *Lactobacillus gasseri*, a form of probiotic strain isolated from human breast milk, was reported to markedly reduce body weight, waist, and hip circumference, BMI, abdominal visceral, and subcutaneous fat areas in obese subjects [52,53]. Probiotics may modulate metabolic derangements especially hyperglycemia via inhibition of intestinal alpha-glucosidases [54].

However, in spite of these positive results, some studies did not find beneficial effects of probiotics in patients with metabolic diseases. Ataie-Jafari reported no advantageous effects of probiotics yoghurt on lipid abnormalities (except total cholesterol) in hypercholesterolemic subjects [55]. Similarly, a recent study found no significant effects of probiotic on the levels of insulin, hs-CRP, interleukin-6, blood glucose, triglycerides, LDL, HDL, and total cholesterol, as well as anthropometric parameters such as body mass index (BMI) and waist to hip ratio in type 2 diabetic patients [56]. These conflicting data might be due to the differences in study design. For instance, the sample size of some of the studies was as small as 14 or 17 [55,56]. While these studies reported no significant effect, a similar and recent study but with bigger sample size showed that consumption of probiotic yoghurt (of comparable composition and dose) markedly reduced the concentrations of CRP, blood glucose, and glycosylated hemoglobin in type 2 diabetic patients [57]. This suggests sample size and other study design-related factors might contribute to the observed lack of statistically significant data. This is evidenced by the non-significant increased level of HDL cholesterol and a declining trend in the levels of hyperinsulinemia, triglycerides, malondialdehyde, and IL-6 [56]. Hence, it is important to consider the differences in study design while interpreting some of these contradictory data.

These studies demonstrate the effects of probiotics in ameliorating metabolic, endocrine, and lipid abnormalities in rodents and humans with metabolic disorders. The lack of beneficial effects of probiotics in subjects with metabolic derangements as reported in some studies may be due to differences in study design. However, it is important to point out that data from clinical studies are limited and further studies are still needed in human subjects with metabolic diseases. The importance of additional clinical studies is reinforced by a study which associates higher counts of gut Lactobacillus species with increased BMI and glycemia in healthy and elderly adults [58].

### 3.2. Modulation with Prebiotics

Besides probiotics, the gut microbiota can be modulated via administration of prebiotics. Prebiotics refer to digestion resistant carbohydrates or food ingredients that resist degradation and absorption in the upper digestive tract and selectively enhance the growth and/or activity of one or a limited number of resident gut microbes that are beneficial to the host [59]. Prebiotic supplementation in obese or diabetic mice markedly lowered fasting plasma glucose, improved glucose tolerance and leptin sensitivity, increased the levels of satiety hormones, reduced low-grade inflammation, plasma triglycerides, and muscle fat content [60,61]. These effects were associated with altered gut microbiota composition (decreased number of Firmicutes and increased abundance of Bacteroidetes) [60,61]. Prebiotic supplementation of high-fat fed mice also decreased body weight gain, adipocyte size, adiposity, serum and hepatic cholesterol levels, and insulin resistance [62,63]. The prebiotic effect of honey, a natural product enriched in oligosaccharides, is well documented [64–66]. Honey has been shown to improve glycemic control and ameliorate metabolic disturbances in diabetic rats [67]. Another prebiotic, inulin, was recently shown to increase bacterial deconjugation of bile acids, increase the villus cell height in the proximal colon, and also increase cecal weight [68]. Laminarin or fucoidan supplementation reduced the population of Enterobacteriaceae and abundance of attaching and effacing *Escherichia coli* strains as well as improved gut barrier functions in pigs. These prebiotics also markedly down-regulated the colonic mRNA expression of proinflammatory cytokines [69]. Table 2 summarizes some of the findings on the effects of prebiotics on the composition of gut microbial community and their metabolic influence in rodents.

In healthy human subjects, consumption of prebiotics results in satiety, reduced energy or food intake, and increased levels of satiety peptides [70]. Prebiotics have been shown to contribute to weight loss and also improve metabolic parameters including insulin resistance in overweight or obese individuals [71]. A recent study showed that type 2 diabetic patients treated with transglucosidase (which generate prebiotic fiber including oligosaccharides from dietary starch in the human GIT) had reduced levels of hyperglycemia and suppressed body weight gain [72]. These effects were mediated via increased gut production of oligosaccharides and alteration of the gut microbiota composition (increased Bacteroidetes-to-Firmicutes ratio) [72]. While certain evidence suggests that prebiotic metabolic products (SCFAs) may aggravate metabolic health [73], there are more compelling findings demonstrating their benefits in metabolic disorders [74,75]. In spite of this, there are few reports suggesting insignificant effect of prebiotics on intestinal microbiota composition in adults with metabolic disorders [76]. Likewise, prebiotic-induced alterations in the composition of the gut microbiota were shown not to contribute to improved whole-body insulin sensitivity in overweight individuals [77]. These conflicting results on the effects of prebiotics on gut microbiota composition and metabolic derangements may be due to several factors, such as differences in soluble and insoluble fiber, as well as fat composition of prebiotics. Baer and colleagues reported that when added to a high fat diet, soluble fiber increased metabolizable energy in humans, whereas it reduced metabolizable energy when added to a low fat diet [78]. Similar observations (increased weight gain) were reported in high fat-induced obese mice fed soluble fiber while the mice showed markedly reduced body weight when fed insoluble fiber [79]. These data seem to indicate that besides the non-digestible carbohydrates, other constituents of prebiotics play a role in altering gut microbiota composition and subsequently improving metabolic disorders.

By and large, these effects of prebiotics clearly suggest that the growth and activity of gut microbiota can be modulated by administration of prebiotics. Considering that prebiotics generally consist of various components, it is essential to investigate which of the constituents play a more predominant role in modulating gut microbiota and the associated improved metabolic derangements. Studies focusing on the differences on the prebiotic effects of soluble and insoluble fiber are also warranted.

### 3.3. Modulation with Antimicrobial Agents

Administration of antimicrobial agents including broad-spectrum antibiotics is another viable option to modulate the gut microbiota. This causes reduction of bacterial biodiversity and prolongation of gut colonization by microbes. The supplementation of obese mice with a combination of two antibiotics, norfloxacin and ampicillin, considerably improved fasting glucose, oral glucose tolerance, and insulin resistance [80]. These improved metabolic parameters were associated with altered gut microbiota and reduced plasma LPS and hepatic triglycerides, as well as enhanced hepatic glycogen storage [80]. In mice fed a high-fat diet or sugars, antibiotic treatment reduced the levels of fasting glucose, insulin, LPS, hepatic lipid and inflammatory markers, such as IL-6 and TNF-α [81,82]. Similarly, administration of subtherapeutic doses of antibiotics (penicillin, vancomycin, penicillin plus vancomycin, or chlortetracycline) to young mice markedly perturbed both the composition and metabolic proficiencies of gut microbiota. This was evidenced by the considerable alterations of microbial taxonomy and important genes involved in the breakdown of carbohydrates to SCFAs in addition to those involved in the regulation of hepatic lipids, cholesterol, and triglyceride metabolism [83]. The antibiotic-treated mice also had increased colonic levels of SCFAs and gastric inhibitory polypeptide (GIP). Other recent studies have also corroborated the beneficial effects of antibiotics on metabolic abnormalities (improved glucose tolerance and reduced levels of fasting blood glucose, triglyceride, TNF-alpha, and decreased weight gain) in obese mice [84,85]. These effects were associated with reduced diversity of gut microbiota [84]. Antibiotic (vancomycin)-treated neonatal NOD mice, a mouse model of diabetes, were shown to have significantly lower diabetes incidence, whereas the antibiotic treatment decreased insulitis and blood glucose levels in adult NOD mice. The antibiotic administration also altered the microbial composition in NOD mice as evident by reduced major genera of Gram-positive and Gram-negative microbes but increased abundance of *Akkermansia muciniphila* [86]. The findings on the modulation of gut microbiota via administration of anti-microbial agents are shown in Table 3.

Similarly, human subjects treated with antibiotics have demonstrated altered gut microbiota. Short-term antibiotic treatment has been shown to reduce bacterial colonization and diversity in adult subjects [90], as well as in neonates [101]. Antibiotic treatment markedly reduced the levels of Bifidobacterium spp. and perturbed the relative abundance of nine other genera including Lactobacillus spp. in the intestine of older adults. Other compositional changes in the intestinal microbiota were also observed [91]. A recent study also reported that antibiotic-treated subjects showed greater and less balanced sugar anabolic capabilities than non-treated individuals. The study found that antibiotic administration changed the active portion of enzymes regulating the thickness, composition, and consistency of the mucin glycans [102]. The impact of long-term administration of antimicrobial agents on the gut microbiota composition and diversity has also been demonstrated [103].

These findings convincingly indicate that the gut microbiota can be modulated via administration of anti-microbial agents. In animal studies, altered gut microbiota, as result of treatment with antibiotics or anti-microbial agents, leads to amelioration of several metabolic derangements including impaired glucose homeostasis and lipid abnormalities. However, in clinical studies majority of the studies focused primarily on the characterization of the composition and diversity of gut microbes. Hence, it remains unclear if anti-microbial induced-gut microbiota alteration in human subjects with metabolic disorders is associated with improvements in metabolic derangements as observed in animal studies. Further studies are, thus, required to address this uncertainty.

### 3.4. Modulation with Surgery

Gut weight loss surgery is performed in certain portions of the GIT (mainly stomach and intestine) in individuals who are morbidly obese to achieve weight loss. The weight loss surgical interventions, generally referred to as bariatric surgery, usually utilized in morbid obesity include sleeve gastrectomy and gastric bypass [104]. Bariatric surgery promotes and sustains weight loss, improves pancreatic β-cell function, enhances insulin sensitivity, reduces adiposity, ameliorates diabetes mellitus and other metabolic parameters, as well as improves cardiovascular risk factors and reduces mortality rate [93,104]. Until now, the mechanisms by which bariatric surgery reduces body weight and fat as well as improves metabolic parameters in obese subjects remained largely indefinite. Evidence now suggests that these health beneficial effects of bariatric surgery may be due to restructured gut microbiota, which in turn alters host-microbial interactions. Roux-en-Y gastric bypass (RYGB) has been shown to alter the gut microbiota as evidenced by the proportional increase of Gammaproteobacteria in post-gastric-bypass surgery individuals [25]. The diets and change in body weight may also play a role on the effect of RYGB on gut microbiota. This is important because a recent study showed that calorie restriction contributes to short-term metabolic benefits of RYGB surgery in obese type 2 diabetic patients [94]. However, it has lately been shown that the population and diversity of gut microbiota can be modulated via RYGB independent of the variation in calorie ingestion [92]. A study that assessed the impact of RYGB on gut microbial composition in morbidly obese patients with type 2 diabetes mellitus found that RYGB induced a rise of Proteobacteria and a decrease of Firmicutes and Bacteroidetes [95]. It was also observed that some of the RYGB-altered bacterial populations were associated with plasma glucose, triglycerides, total- or LDL cholesterol [95]. Similar to previous findings, RYGB was reported to increase the abundance of Proteobacteria and alter several bacterial genera and white adipose tissue (WAT) genes. A correlation between variations in bacterial genera and metabolic parameters and altered WAT gene expression was also found. The effect of RYGB surgery in shifting the gut microbiota has also been demonstrated in rodents [105]. A recent study provides direct and compelling evidence in support of the role of weight loss surgery in the modulation of gut microbiota. Liou and colleagues showed that RYGB in mice altered gut microbiota (similar to the microbial alterations observed in human patients following gastric bypass) leading to a considerable increase in the abundance of certain bacterial genera, Escherichia and Akkermansia [106]. These findings are important because a recent study showed that treatment with *Akkermansia muciniphila* markedly attenuates high-fat diet-induced metabolic aberrations, such as insulin resistance, endotoxemia, excessive fat deposition and adipose tissue inflammation [107]. Supplementation with this microbe also increases gut barrier, gut peptide secretion, and intestinal levels of endocannabinoids, important immunodulatory and anti-inflammatory molecules [107]. A recent review of literature reveals that RYGB-modified gut microbiota is uniquely different from the gut microbial alterations following weight loss without RYGB surgery [108].

These findings reveal that bariatric surgery is not only associated with altered composition and diversity of gut microbiota, but also improved metabolic abnormalities. Bariatric surgery can, thus, be considered one of the effective ways to modulate the gut microbiota in the treatment of metabolic diseases.

### 3.5. Modulation with Weight Loss Strategies

Intervention strategies, such as calorie restriction, exercise and other behavioral or life style changes, which enhance weight loss are known to be beneficial in the management of metabolic diseases [109]. Available evidence suggests that these beneficial effects may be mediated via gut microbiota. Obese subjects supplemented with a high protein and low carbohydrate diet for four weeks had significantly reduced total fecal SCFAs compared with when supplemented with higher carbohydrate content. These data correlated with significantly reduced abundance of butyrate-producing bacteria, *Eubacterium rectale*, Roseburia spp. and bifidobacteria [96,97]. A study that evaluated the effect of calorie-restricted diet combined with increased physical activity in overweight adolescents found the intervention markedly increased bacterial counts of *Bacteroides fragilis* and Lactobacillus groups while it decreased the counts of *Bifidobacterium longum* and *Bifidobacterium adolescentis* groups. It was also found that a greater weight loss (>4.0 kg and resulting in significant reductions of BMI and BMI z-score) was associated with marked alterations of gut microbes compared to less weight loss (<2.0 kg) [99]. Similar effects of considerable weight loss on gut bacteria were also reported by Nadal and co-workers. The authors found a significant correlation between reduced proportions of *Clostridium histolyticum* and *E. rectale-C. coccoides* and weight loss in overweight and obese adolescent population. Compared to less weight loss (<2.5 kg), greater weight loss (>4.0 or 6.0 kg) resulted in more loss of total fecal energy and bacterial proportions [100]. The beneficial effects of calorie restriction on the gut microbiota including reduced levels of lipopolysaccharide-binding protein have also been demonstrated in rodents [110]. Vegetarian diet is rich in fiber and is commonly recommended as a component of lifestyle intervention to promote weight loss [111]. A recent study found that strict vegetarian diet ameliorates several abnormalities associated with metabolic diseases. These include improved fasting and postprandial glycemia, decreased body weight and reduced concentrations ofHbA1c, total cholesterol, LDL-C, and triglycerides [98]. These effects are mediated via modulation of gut microbiota (reduced Firmicutes-to-Bacteroidetes ratio).

There is no doubt that studies which have investigated the effects of weight loss/calorie restriction on gut microbiota composition are still limited. It is however interesting to note that these few available data clearly suggest that gut microbiota can be modulated via interventions that target weight loss. These approaches, which include calorie restriction, consumption of vegetarian diet, and increased physical activity still need additional studies. Some of the key strategies which have been used to modulate the human gut microbiota and their outcomes are presented in Table 4.

## 4. Modulation of Gut Microbiota in the Management of Metabolic Disorders: The Challenges

Over the past couple of years, research has continued to delineate the gut microbiota. This is made possible as a result of bacterial cultivation and culture-independent molecular techniques such as genome sequencing, genetic fingerprinting, metagenomics, metabolomics, metaproteomics, metatranscriptomics, and proteogenomics. This has led to the identification and more in-depth characterization of phylogenetic and functional diversity of gut microbial communities [112]. From the plethora of data that emerged from these techniques, it is now obvious that the gut microbiota interacts with its host and influences host’s metabolism, which may contribute to metabolic diseases. However, in spite of numerous published data linking the gut microbiota to metabolic diseases, a number of challenges (issues or questions) still exist. These challenges, which need to be addressed, relate to how the gut microbes modulate, influence and interfere with the host’s mechanisms to elicit systemic metabolic effects. These gaps in our understanding, as highlighted below, constitute the main challenges to successfully target the gut microbiota in the management of metabolic disorders.

Considering that the gut microbiota composition and diversity within a population or individuals change over time, studies that investigate the gut microbial profile with increasing age are vital. It would also be profound to have studies that characterize the profile of a normal microbiota from that seen in diseased state. Besides, it would be interesting to have data on the gut microbiota composition and diversity before the development of metabolic diseases. Comparison of such data with those obtained following the development of metabolic diseases will in no doubt be insightful. As demonstrated previously, certain microbes, such as *Akkermansia muciniphila*, has been identified as one of those bacterial species that play a major role in controlling or preventing obesity and metabolic disorders [86,107]. Hence, the data may help in identifying the most predominant microbes at the different stages of development or life. With the aid of new culture-independent techniques, such findings may be used to develop vigorous predictive biomarkers or individual’s risk for metabolic diseases. The availability of such data may also help in selecting a particular or combination of bacterial genus/genera, species or strains for the manipulation of gut microbiota in the treatment of metabolic diseases. Such information may also be used to assess the effectiveness and efficacy of therapy or interventions. However, such studies may be challenging considering that certain host factors, such as genotype and environmental factors including diets are known to influence microbial populations [113].

The relative proportion of some major phyla of gut bacteria, such as Bacteroidetes and Firmicutes (a lower proportion of Bacteroidetes and higher abundance Firmicutes), has been associated with metabolic diseases such as obesity [114]. On the other hand, there is scanty of studies that have investigated the potential causal role of these bacterial phyla in metabolic diseases. In other words, there are more studies associating the gut bacterial phyla with metabolic diseases than there are implicating the role of these bacterial phyla in the etiology or progression of metabolic diseases. It remains uncertain whether by nature, leanness, or maintenance of normal body weight preferentially enhances the growth and activity of certain beneficial bacteria (such as Bacteroidetes) instead of harmful bacteria, such as Firmicutes. Could this be part of inherent mechanisms employed by the body to prevent metabolic diseases such as obesity? Hence, due to dearth of data, it remains unclear if this unusual bacterial phyla abundance plays a role in the etiology of metabolic diseases or is a consequence of these diseases. Hence, more rigorous studies that focus on these two important phyla of gut bacteria in both mice and humans are necessary. Such research may include studies investigating if administration of some beneficial bacteria (belonging to the phylum Bacteroidetes) to obese mice or high-fat fed mice prevents the development of obesity. Likewise, bacterial species belonging to the phylum Firmicutes can be administered to healthy mice fed normal diet or germ-free mice. Data from such study designs will be very insightful and may help to answer the important question of whether certain bacteria (Bacteroidetes or Firmicutes) can cause or prevent metabolic disorders such as obesity. Besides, certain bacterial species, such as *L. reuteri*, are found in large quantities in obesity while others, such as *B. animalis*, are associated with normal weight [23]. Within the genus Lactobacillus, *L. reuteri* is associated with obesity, whereas *L. gasseri* and *L. plantarum* are found in lean or normal weight subjects. This suggests that the gut microbiota composition is linked to normal body weight and obesity at the species. Thus, research that focuses on investigating the role of bacteria at species and strain levels in metabolic disorders are highly desirable. Targeting some of these bacterial species with specific antibiotics may also be of therapeutic benefits.

At present, a large proportion of the data linking the gut microbiota to metabolic diseases are derived from diet-induced rodents. To further unravel the role of gut microbiota in metabolic diseases, it seems logical to utilize other animal models of metabolic diseases, such as drug-induced diabetes in both germ free and conventionally raised mice. These models will provide insight on the influence of gut microbiota in the etiology or progression of metabolic diseases or *vice versa*. These models will also reveal if systemic metabolic disturbances such as hyperglycemia and lipid abnormalities (for instance, in STZ-induced diabetic germ free and conventionally raised mice) could alter the composition and diversity of gut microbiota. If it does, it would be remarkable to know or identify which of the bacterial phyla, genera, species, or strains are altered.

The gut microbiota is known to play an important role in the biotransformation of xenobiotics [115]. The type of diet (whether vegetarian diet or Western diet) may alter the gut microbiota composition [116]. In particular, Western diet is enriched in protein and most constituents are heat-processed. It is known that thermal processing of food promotes the formation of Maillard reaction products (MRPs) [117]. The MRPs, formed by reaction between food protein and sugar, are digestion-resistant in the small intestine and hence are substrates for colonic gut microbes [118]. Evidence indicates that MRPs can modify colonic microbial composition and may also be involved in inflammatory responses systemically [119]. Currently, not much is known about the fate of MRP metabolism by the gut microbes. Could these MRPs influence and enhance the activity of harmful colonic bacteria? What about the end-products of MRP metabolism by colonic bacteria and their potential systemic pathological effects? Those questions become relevant in view of a recent finding that indicates gut microbes metabolize *L*-carnitine (which is predominant in red meat) and the end-product enhances the development of atherosclerosis [120]. Similarly, it was recently discovered that gut microbial metabolism of the choline moiety in dietary phosphatidylcholine generates trimethylamine-*N*-oxide, which is a proatherosclerotic metabolite [121]. Considering these findings, studies that investigate the systemic effects of end products derived from gut microbial metabolism of various diets—such as Western diets or high fat/fructose diet in the pathophysiology of metabolic disorders are necessary.

With regards to modulating the gut microbiota via gastric bypass surgery, the evidence is compelling. While previous findings revealed that RYGB surgery caused a shift of obese microbiota profile towards that observed in normal weight animals and individuals, it was uncertain whether the altered gut microbiota was a primary consequence of RYGB or its secondary effect or due to other factors such as calorie restriction or weight change. However, a research finding published lately seems to have addressed, in part, this ambiguity. The researchers reported that non-operated, germ-free mice transferred with the gut microbiota from RYGB-treated mice were characterized by marked weight loss and decreased fat mass [106]. On the one hand, this study provides convincing evidence in support of a role of altered gut microbiota in the health beneficial effects of RYGB surgery on body weight and metabolic abnormalities. On the other hand, it also reveals that the greatest changes in the microbiota occurred in the distal end of the GIT, which is far from the surgical site. This is really an interesting finding because it raises some concerns and questions. For instance, could it be that there is a communication link or pathway between the RYGB surgical site and distal gut? Or could it be that the RYGB surgery influences a major systemic pathway that controls the distal gut microbiota? These are questions begging for answers and can only be addressed with further investigations.

Potential modulation approaches of gut microbiota, such as prebiotics, probiotics, and antibiotics are associated with some restrictions. These limitations include the problems of viability and sustainability of ingested bacteria (probiotics) in the gut. Others include the host-specificity of probiotics, which limits the generalization of health effects attributed to probiotics. On the other hand, the beneficial effects of prebiotics are often short-lived and also liable to be surpassed or countered by diets. Studies comparing, both individually and in combined form, the various bacteria commonly used as probiotics are vital. Interventions involving the comparison of combination of prebiotics and probiotics *versus* either agent are also crucial. With regards to modulating the gut microbiota via antimicrobial agents, one of the major setbacks is the lack of bacterial species specificity. The use of antimicrobial agents may also target beneficial microbial populations [122]. Besides, while short-term use of these agents is advantageous, the same cannot be said for their long-term use. Prolonged administration of antimicrobial agents may contribute to the spread of resistant bacteria and resistance genes [123]. This may in turn results in reduced effectiveness and efficacy caused by microbial resistance. Recent findings reveal that modulation of gut microbiota via antibiotics also carries the potential to exacerbate metabolic disorders by increasing adiposity and body mass index [83,124]. These are areas of research that need further investigations.

Current research is yet to answer the question of how the gut microbiota modulates host’s metabolism. This is a research area that needs to be addressed. This is important because evidence seems to suggest that the gut microbiota can communicate with the host’s system (such as central nervous system) by modulating the endocrine, immune, and neural pathways. This has been shown to control several organs and functions including behavioral and cognitive functions of its host [125,126]. Therefore, identifying the potential bioactives, molecules, pathways, or even genes, which may serve as links between the gut microbes and host is imperative. This is relevant in view of the discovery of Toll-like receptors (TLRs), which are involved in microbiota-host cross talk. Several roles, such as maintenance of gut microbiota homoeostasis and synthesis of antimicrobial peptides have been attributed to these TLRs (especially TLR2 and TLR4) [127]. With the discovery of such mechanisms, it may become unnecessary to modulate directly the gut microbes. Instead, the identified links, pathways or molecules may serve as therapeutic targets, which may lead to synthesis or development of inhibitors or modulators of these pathways or molecules. This will also help to bypass some of the limitations (as highlighted) of direct modulation of gut microbiota. Other areas that need to be addressed include elucidating the mechanisms by which harmful microbes impair normal metabolism in the host, as well as the interactions between beneficial microbes and the host.

Future research should also be directed towards personalized modulation of gut microbiota. This will entail identifying food ingredients capable of inhibiting and enhancing the growth of harmful and beneficial microbes, respectively. Besides, the type of food consumed is known to influence the gut microbiota composition and diversity [128]. It is, however, unclear if the gut microbiota influences the choice of foods. Additional studies that take into cognizance the impact of genetic and environmental factors on the composition of the microbiota are also vital. These are areas that require further research in order to successfully target the gut microbiota in the management of metabolic disorders.

## 5. Conclusions

There is no doubt that research investigating the role of gut microbiota in metabolic diseases is still in its infancy. While the prospect of modulating the gut microbiota seems promising, however, there is a need for more rigorous *in vitro*, animal, and clinical studies. With further research that addresses some of the aforementioned challenges, there is no doubt we would better understand how the gut microbiota contributes to metabolic diseases. Better understanding of the role of gut microbiota in interfering with the host’s metabolism will invariably contribute to improved and more successful modulation of the gut microbiota in the treatment of metabolic diseases. This research is necessary because it will be helpful to pinpoint the exact interactions or underlying mechanisms of gut microbes with their host.

In addition, even though modulation of gut microbiota appears to be an attractive strategy for the treatment of metabolic diseases including obesity and diabetes mellitus, it is important to note that the pathogenesis of metabolic diseases is not exclusively limited to the role of gut microbiota alone. Metabolic diseases are caused by many factors including hereditary, increased consumption of energy-rich diets and reduced or lack of physical activity. Hence, the modulation of gut microbiota, if successful, may also serve as adjunct to current treatments of metabolic diseases.

### Review Criteria

PubMed was searched using the following key words (each key word alone and in combination): “gut microbiota”, “microbiome”, “microbe”, “microorganism”, “prebiotic”, “probiotic”, “antimicrobial agent”, “antibiotic”, “bariatric surgery”, “weight loss”, “metabolic disease”, “metabolic disorder”, “insulin resistance”, “diabetes mellitus” and “obesity”. The search was also performed with the plural form of the words (e.g., microbe, microbes; prebiotic, prebiotics). Google scholar search was also carried out to identify additional relevant articles. Both original and review articles were considered. Only articles in English language were included. The bulk of the literature search was carried out in February and March 2013 and updated in October 2013. Additional articles were added following peer reviewers’ suggestions.

## Figures and Tables

**Table 1.** Probiotic modulation of gut microbiota in rodents.

Reference	Probiotic administered	Rodent/metabolic model	Study design (including treatment, dosage and duration)	Effects on gut microbiota	Effects on metabolic derangements
Hsieh *et al.* [39]	*Lactobacillus reuteri*	Rats; insulin resistance	Rats fed a high-fructose diet with *L. reuteri* at a dose of 2 × 10^9^ CFU/rat administered daily for 14 weeks	↑ numbers of Bifidobacterium and Lactobacillus species. ↓ number of Clostridium species	↓ Serum levels of insulin, leptin and C-peptide, ↓ Serum levels of glucose, HbA1c and glucose intolerance ↓ Serum LDL-C, TG and TC ↓ Serum levels of AST and ALT ↑ Serum GLP-1 ↓ Adipose tissue concentrations of IL-6 and TNF-α ↓ Elvol6, SREBP-1c and FAS ↑ Hepatic AEs (GR and SOD)
Yadav *et al.* [43]	*Lactobacillus acidophilus; Lactobacillus casei*	Rats; Diabetes mellitus	STZ-induced diabetic rats treated with dahi containing *L. acidophilus* and *L. casei* (15 g/rat/day) for 4 weeks	Uncharacterized	↓ Plasma glucose, TG, LDL-C, TC and LDL/HDL ratio ↑ Plasma insulin and HDL-C ↑ Hepatic glycogen. ↓ Hepatic TC and TG ↓ Panccreatic OD ↑ Pancreatic AEs (SOD, CAT and GPx)
Zhang *et al.* [46]	*Lactobacillus casei Zhang*	Rats; Hyperinsu-linemia; Impaired glucose intolerance	Rats fed fructose water and treated with *L. casei Zhang* at a dose of 1 × 10^9^ CFU/day/rat	↑ numbers of *Bacteroides fragilis* and Bifidobacterium & Lactobacillus species. ↓ number of Clostridium species	↑ Glucose tolerance ↓ Serum MDA ↓ Serum insulin and GLP-2 ↑ Hepatic expression of adipoR2, LXR-α and PPAR-γ ↓ Hepatic glycogen ↑ Intestinal bile acids
Bejar *et al.* [41]	*Lactobacillus plantarum*	Rats; Diabetes mellitus	Alloxan-induced diabetic rats treated with *L. plantarum*	Uncharacterized	↓ serum levels of plasma glucose, triglyceride, LDL cholesterol, LDL/HDL ratio, creatinine, urea and transaminases. ↑ HDL cholesterol ↓ hepatic total cholesterol and triglycerides ↓ pancreatic and plasmatic lipase activities ↓ pancreatic β-cell, renal and hepatic injuries
Park *et al.* [42] and Sakai *et al.* [40]	*Lactobacillus plantarum*	Mice; obesity	Mice were fed a high-fat diet and administered 1 × 10^7^ or 1 × 10^9^ CFU/mouse of *L. plantarum* or *L. rhamnosus* daily for 12 weeks	Uncharacterized	↓ Serum levels of TG, insulin and leptin ↓ levels of glucose and non-esterified fatty acids ↓ mRNA expression of adipose tissue IL-1β ↓ levels of back and epididymal fat ↓ triglyceride, insulin and leptin ↑ hepatic mRNA expression of PPARα and CPT-I ↓ hepatic mRNA expression of ACC, SREBP-1 and LXRα ↓ Epididymal adipose tissue PPARγ expression
Kim *et al.* [45]	*Lactobacillus rhamnosus*	Mice; obesity	Mice were fed a high-fat diet and administered 1 × 10^9^ CFU/mouse of *L. plantarum* or *L. rhamnosus* daily for 13 weeks	Uncharacterized	↓ weight gain ↑ insulin sensitivity and adiponectin secretion ↑ Adiponectin production ↑ Expression of hepatic fatty acid oxidative genes ↓ Expression of gluconeogenic genes ↑ mRNA expression of skeletal GLUT4
Kang *et al.* [44]	*Lactobacillus gasseri*	Mice; obesity	Mice fed high-sucrose diet and *L. gasseri* (1 × 10^9^ or 10^10^ CFU/mouse) for 10 weeks	Uncharacterized	↓ body weight and white adipose tissue weight ↓ serum levels of insulin and leptin ↑ mRNA levels of GLUT4 and fatty acid oxidation-related genes (ACO, CPT1, PPARα and PPARδ) ↓ mRNA levels of fatty acid synthesis-related genes (SREBP-1c and ACC)

HbA1c, glycated hemoglobin; LDL-C, low-density lipoprotein cholesterol; HDL-C, high-density lipoprotein cholesterol; TG, triglycerides; TC, total cholesterol; AST, aspartate aminotransferase; ALT, alanine transaminase; GLP-1, glucagon-like peptide 1; IL-6, interleukin 6; TNF-α, tumor necrosis factor alpha; Elvol6, fatty acid elongase 6; SREBP-1c, sterol regulatory element-binding protein 1c; FAS, fatty acid synthase; OD, oxidative damage; AEs, antioxidant enzymes; GR, glutathione reductase; SOD, superoxide dismutase; CAT, catalase; GPx, glutathione peroxidase; MDA, malondialdehyde; AdipoR2, adiponectin receptor 2; LXR-α, liver X receptor-alpha; PPAR-γ, peroxisome proliferator-activated receptor gamma; PPAR-α, peroxisome proliferator-activated receptor alpha; PPARδ, peroxisome proliferator-activated receptor delta; CPT1, carnitine palmitoyl-transferase I; ACO, acyl CoA oxidase; UCP3, uncoupling proteins3; GLUT4, glucose transporter 4; SREBP-1c, sterol regulatory element-binding protein-1c; ACC, acetyl-CoA carboxylase; ↑ = Increase/enhance; ↓ = Reduce/suppress.

**Table 2.** Prebiotic modulation of gut microbiota in rodents.

Reference	Rodent/metabolic model	Study design (including treatment, type of prebiotics, dosage and duration)	Effects on gut microbiota	Effects on metabolic derangements or abnormalities
Dewulf *et al.* and Neyrinck *et al.* [62,63]	Mice; Obesity	Mice fed high-fat diet and provided with inulin-type fructans (0.2 g/day per mouse) or arabinoxylans (10% *w*/*w*) for 4 weeks	↑ Abundance of Bifidobacteria and Bacteroides-Prevotella species ↓ Number of Clostridium and Roseburia species	↓ Body weight gain ↓ Serum and hepatic cholesterol levels ↓ Insulin resistance ↓ Adipocyte size and adiposity ↓ PPAR-γ-activated differentiation factors ↑ adipose tissue GPR43 expression ↑ Gut barrier function ↓ Inflammatory markers ↓ Expression of genes involved in fatty acid uptake, differentiation, fatty acid oxidation and inflammation ↓ Lipogenic enzyme activity
Everard *et al.* [60]	Mice; Obesity and diabetes mellitus	Genetic or diet-induced obese and diabetic mice fed with prebiotic-enriched diet (oligofructose, 0.3 g/mouse/day) for 8 weeks	↑ Bacteroidetes phylum ↓ Firmicutes phylum	↓ Glucose intolerance and plasma glucose ↑ L-cell number and plasma GLP-1 ↑ Leptin sensitivity ↑ LPL and proglucagon mRNA levels ↓ Plasma TG and adipose tissue weight ↓ Muscle TG and lipid content ↓ Adipose tissue oxidative stress ↓ Low grade inflammation
Parnell and Reimer [61]	Rats; Obesity	Obese rats fed 10% and 20% fibre (inulin: oligofructose) diets for 10 weeks	↑ Number of Bacteroidetes group ↓ Number of Firmicutes and *Clostridium coccoides* group ↑ Bifidobacterium and Lactobacillus species	↓ Energy intake ↓ Glucagon ↑ GLP-1 secretion ↓ Ghrelin response ↑ mRNA levels of caecal peptide YY and proglucagon ↓ Ghrelin O-acyltransferase mRNA levels
Erejuwa *et al.* and Nemoseck *et al.* [67,87–89]	Rats; Diabetes mellitus	Rats fed honey or sucrose as well as STZ-induced diabetic rats fed honey for 33 days	Uncharacterized	↓ Body weight gain in honey fed *vs*. sucrose fed rats ↑ Body weight in honey-treated STZ *vs*. STZ control rats ↓ Epididymal fat weight ↓ Serum levels of glucose and fructosamine ↓ Serum levels of TG, VLDL-C, leptin and bilirubin ↑ Serum albumin, insulin and HDL-C

GPR, G-protein-coupled receptor; PPAR-γ, peroxisome proliferator-activated receptor gamma; GLP-1, glucagon-like peptide 1; LPL, lipoprotein lipase; TG, triglycerides; HDL-C, high-density lipoprotein cholesterol; VLDL-C, very low-density lipoprotein cholesterol; STZ, streptozotocin; ↑ = Increase/enhance; ↓ = Reduce/suppress.

**Table 3.** Antimicrobial modulation of gut microbiota in rodents.

Reference	Antibiotics administered	Rodent/metabolic model	Study design (including treatment, dosage and duration)	Effects on gut microbiota	Effects on metabolic derangements
Membrez *et al.* [80]	Norfloxacin and ampicillin	Mice; Insulin resistance and obesity	Genetically obese, diet-induced obese and insulin-resistant mice treated with norfloxacin and ampicillin (1 g/L each) for 14 or 17 days	Uncharacterized	↓ Blood glucose and glucose intolerance ↓ Plasma insulin and insulin resistance ↓ Plasma LPS ↓ Hepatic TG ↑ Adiponectin ↑ Hepatic glycogen storage
Carvalho *et al.*, Bergheim *et al.* and Cho *et al.* [81–83]	Aampicillin, neomycin and metronidazole	Mice; Fatty liver, adiposity	Mice fed HFD with ampicillin, neomycin and metronidazole, each at 1 g/L) or polymyxin B (92 mg) and neomycin (216 mg) for 8 weeks	↓ Total bacterial count ↓ Bacteroidetes and Firmicutes	↓ Food intake and body weight gain ↓ Plasma glucose and insulin ↑ Glucose and insulin tolerance ↓ LPS, TNF-α and IL-6 ↓ TLR4, JNK, IKKbeta ↑ Colonic levels of SCFAs and GIP ↑ Phosphorylation of IR, IRS-1 and Akt ↑ Circulating acetate ↑ AMPK phosphorylation
Murphy *et al.* and Bech-Nielsen *et al.* [84,85]	Vancomycin, ampicillin	Mice; Obesity	Mice fed a low-fat or high-fat diet with/without vancomycin (2 mg/day) for 8 weeks	↑ Proteobacteria ↓ Bacteroidetes and Firmicutes	↓ Body weight gain ↑ Glucose tolerance ↓ Blood glucose ↓ Plasma TG and TNF-α
Hansen *et al.* [86]	Vancomycin	Mice; Diabetes mellitus	NOD mice treated with vancomycin (83 mg/kg/day) until development of diabetes or weaning (28 days)	↓ Bacteroidetes and Firmicutes	↓ Blood glucose ↓ Insulitis score

LPS, lipopolysaccharides; TG, triglycerides; IL-6, interleukin 6; TNF-α, tumor necrosis factor alpha; TLR-4, Toll-Like Receptor-4; JNK, c-Jun N-terminal kinase; IKKbeta, inhibitory-kappaB kinase (IKK)-beta; SCFAs, short chain fatty acids; GIP, gastric inhibitory polypeptide; IR, insulin receptor; IRS-1, insulin receptor substrate 1. ↑ = Increase/enhance; ↓ = Reduce/suppress.

**Table 4.** Modulation of gut microbiota in humans.

Reference	Intervention/modulation	Metabolic disorder	Study design	Effects on gut microbiota and metabolic derangements including other alterations
Andreasen *et al.* and Asemi *et al.* [47,48]	Probiotic *(L. acidophilus* and *B. animalis*)	Insulin resistance; Diabetes mellitus	Randomized, double-blind, controlled studies. 45 males with type 2 diabetes, impaired or normal glucose tolerance treated with/without *L. acidophilus* for 4 weeks and controlled clinical trial; 70 pregnant women given *a probiotic yoghurt containing L. acidophilus* and *B. animalis* (200 g/day) for 9 weeks	↑ *L. acidophilus* ↑ Insulin sensitivity in probiotic group ↓ insulin resistance
Ejtahed *et al.* [49,50]	Probiotic *(L. acidophilus* and *B. lactis)*	Diabetes mellitus	Randomized, double-blind, controlled trials. 60–64 patients with type 2 diabetes mellitus consumed probiotic/non-prebiotic yogurt containing *L. acidophilus* and *B. lactis (*300 g/day*)* for 6 weeks	Gut microbiota: Uncharacterized ↓ Blood glucose and HbA1c ↓ TC and LDL-C in probiotic group ↓ Atherogenic indices (TC:HDL-C ratio and LDL-C:HDL-C ratio) ↑ Erythrocyte TAS, SOD and GPx activities ↓ Serum MDA
Kadooka *et al.* and Jung *et al.* [52,53]	Probiotic (*L. gasseri*)	Overweight and Obesity	Randomized, multicenter, double-blind, placebo-controlled trial. 57 or 87 obese subjects received fermented milk containing *L. gasseri* or without (200 g/day) for 12 weeks	↓ Body weight ↓ BMI ↓ Abdominal visceral, subcutaneous and total fat areas in prebiotic group ↓ Waist and hip circumferences ↓ Waist-to-hip ratio
Asemi *et al.* [51]	Probiotic and prebiotic. Probiotic (*L. acidophilus*, *L. casei*, *L. rhamnosus*, *L. bulgaricus*, *B. breve*, *B. longum*, *S. thermophilus*) Prebiotic (fructo-oligosaccharide)	Diabetes mellitus	Randomized, double-blind, placebo-controlled clinical trial. 54 diabetic patients ingested a multispecies probiotic/non-prebiotic supplement (consisting of *L. acidophilus*, *L. casei*, *L. rhamnosus*, *L. bulgaricus*, *B. breve*, *B. longum*, *S. thermophilus* and fructo-oligosaccharide) for 8 weeks	Gut microbiota: Uncharacterized ↓ Blood glucose increments ↓ Insulin resistance ↓ Serum hs-CRP ↑ Plasma GSH levels
Cani *et al.* and Parnell and Reimer [70,71]	Prebiotic (oligofructose or a mixture of glucosyl- (fructosyl)n-fructose and (fructosyl)mfructose extracted from chicory roots)	Healthy, overweight and obesity	Randomized, double-blind, parallel, placebo-controlled trial 10 healthy adults given 16 g of prebiotics/day	↑ Marker of gut microbiota fermentation (breath-hydrogen excretion) ↓ Body weight ↓ Caloric intake ↓ Plasma glucose and postprandial glucose responses ↓ Insulin levels ↑ Levels of GLP-1 and peptide YY ↓ Ghrelin levels
Sasaki *et al.* [72]	Prebiotic (transglucosidase)	Healthy and Diabetes mellitus	Randomized, double-blind, parallel, placebo-controlled study. 60 diabetic patients received 300 or 900 mg/day of transglucosidase for 12 weeks.	↑ Bacteroidetes-to-Firmicutes ratio ↓ Body weight ↓ Blood glucose
Jernberg *et al.* and O’Sullivan *et al.* [90,91]	Antibiotics (Clindamycin)	Healthy adults	42 elderly subjects were treated with one antibiotic within 1 month	↓ Bacteroides ↓ Bifidobacterium spp. Metabolic derangements: Unevaluated
Zhang *et al.* And Kong *et al.* [25,92]	Gastric bypass	Obesity	Comparison of the structures of microbes in individuals with normal weight, morbid obesity and post-gastric-bypass surgery	↓ Firmicutes ↑ Proteobacteria ↑ Alterations of WAT genes ↑ Associations between gut microbiota composition and WAT gene expression Metabolic derangements: Unevaluated
Kashyap *et al.* [93–95]	Bariatric surgery	Obesity and Diabetes mellitus	Randomized, prospective, controlled and nonrandomized, controlled observational trials Type 2 diabetic subjects with moderate obesity received bariatric surgery Changes of gut microbial composition 3 months before and after RYGB in morbidly obese patients with type 2 diabetes mellitus	↑ Proteobacteria ↓ Firmicutes and Bacteroidetes ↑ Weight loss ↓ Blood glucose ↓ HbA1c ↑ Pancreatic β-cell function ↑ Insulin sensitivity ↓ Adiposity ↑ GLP-1 and peptide YY levels ↓ Ghrelin levels
Duncan *et al.* [96,97]	Weight loss/Caloric restriction	Obesity	Obese and non-obese individuals under conditions of weight maintenance, and undergoing weight loss on reduced carbohydrate diets for 4 weeks	↓ Total fecal SCFAs ↓ Abundance of butyrate-producing bacteria such as Firmicutes, Bifidobacteria, Eubacterium rectale and Roseburia spp. Metabolic derangements: Unevaluated
Kim *et al.* [98]	Weight loss/Caloric restriction/Vegetarian diet	Obesity and Diabetes mellitus	Obese individuals with type 2 diabetes and/or hypertension assigned to a vegetarian diet for 1 month	↓ Firmicutes-to-Bacteroidetes ratio ↓ Body weight ↓ Fasting and postprandial glucose ↓ HbA1c ↓ TC, LDL-C and TG
Santacruz *et al.* and Nadal *et al.* [99,100]	Weight loss, caloric restriction or increased physical activity	Obesity	Longitudinal intervention study Overweight and obese individuals placed on a calorie-restricted diet and increased physical activity program for 10 weeks	↑ Weight loss ↓ BMI and BMI z-score ↑ Total bacteria, Bacteroides-Prevotella group and Lactobacillus group counts ↓ Clostridium coccoides group, Bifidobacterium longum, and Bifidobacterium adolescentis counts Reduced body weight and BMI z-score correlated with reduction of certain gut microbes

HbA1c, glycated hemoglobin; LDL-C, low-density lipoprotein cholesterol; HDL-C, high-density lipoprotein cholesterol; TG, triglycerides; TC, total cholesterol; SOD, superoxide dismutase; GPx, glutathione peroxidase; MDA, malondialdehyde; TAS, total antioxidant status; GSH, glutathione; BMI, body mass index; hsCRP, high-sensitivity C-reactive protein; SCFAs, short chain fatty acids; WAT, white adipose tissue; ↑ = Increase/enhance; ↓ = Reduce/suppress.
